# The benefit of exercise rehabilitation guided by 6-minute walk test on lipoprotein-associated phospholipase A2 in patients with coronary heart disease undergoing percutaneous coronary intervention: a prospective randomized controlled study

**DOI:** 10.1186/s12872-021-02430-7

**Published:** 2022-04-17

**Authors:** Xiangyang Liu, Wanming Zhou, Wenmao Fan, Aihua Li, Jungang Pang, Zefang Chen, Xiangmei Li, Xiulian Hu, Yanmin Zeng, Liangqiu Tang

**Affiliations:** Department of Cardiology, Yuebei People’s Hospital Affiliated To Shantou University, Shaoguan, 512026 Guangdong China

**Keywords:** 6-min walk test, Exercise rehabilitation, Coronary heart disease, Lipoprotein phosphorus esterase A2

## Abstract

**Background:**

Lipoprotein-associated phospholipase A2 (Lp-PLA2) has been taken as a biomarker of inflammation in patients with acute coronary diseases. Regular exercise rehabilitation could attenuate inflammation and promote the rehabilitation of coronary heart disease (CHD). The level of Lp-PLA2 is negatively correlated with 6-min walk test (6-MWT). The exercise prescription of appropriate intensity is the basis of exercise rehabilitation. 6-MWT is associated with maximal oxygen consumption, and can be used to determine the intensity of exercise prescription guiding patients how to do exercise rehabilitation. The aim of this study was to observe the benefit of 6-MWT guided exercise rehabilitation on the level of Lp-PLA2 in patients with CHD undergoing percutaneous coronary intervention (PCI).

**Methods:**

We prospectively, consecutively enrolled 100 patients between Dec 2018 and Dec 2020 in the fourth ward of the Department of Cardiology, Yuebei People's Hospital Affiliated to Shantou University. Eligible patients were 1:1 divided into Group A, with no exercise rehabilitation, and Group B, with regular exercise rehabilitation, using random number table method of simple randomization allocation. Clinical data such as general information, the profile of lipids and the level of Lp-PLA2 were collected at baseline and at 12-week follow-up.

**Results:**

There were no statistically significant differences of the percentages of gender, hypertension, type-2 diabetes mellitus (T2DM), the profile of lipids and level of Lp-PLA2 between the groups at baseline (P > 0.05). The level of Lp-PLA2 decreased at 12-week follow-up, moreover, the decline of the Lp-PLA2 level in Group B was more significant than that in Group A (t = 2.875, P = 0.005). Multivariate linear regression analysis indicated that exercise rehabilitation was independently correlated with the level of Lp-PLA2 (β′ = − 0.258, t = − 2.542, P = 0.013).

**Conclusion:**

Exercise rehabilitation for 12 weeks guided by 6-MWT can further reduce the level of LP-PLA2 in patients with CHD undergoing PCI.

*Trial registration* This trial was registered on the Chinese Clinical Trial Registry: ChiCTR2100048124, registered 3 July 2021- Retrospectively registered. The study protocol adheres to the CONSORT guidelines.

## Background

With the change of environment, development of social economy and the increase of social pressure, the number of patients with coronary heart disease (CHD) has been increasing continuously. Although reascularization technology of CHD continues to progress and new drugs emerge in endlessly, mortality caused by the disease itself and the related complications is still increasing. Inflammation plays an important role in CHD [1, 2], and has been taken as a therapeutic target in atherosclerosis [3]. At the same time, exercise intervention could attenuate inflammation and inflammatory damage [4, 5]. Lipoprotein-associated phospholipase A2 (Lp-PLA2), a biomarker of inflammation, is produced mainly by vascular endothelial cells and macrophages from atherosclerotic plaques [6], which was associated with unstable plaque or acute thrombosis. Previous study has shown that Lp-PLA2 is an independent indicator for incident cardiovascular diseases [7] and the severity of coronary lesion [8–10]. And Darapladibas, a selective reversible inhibitor of Lp-PLA2, could inhibit plasma Lp-PLA2 activity and reduce the levels of inflammatory biomarkers, such as IL-6 and hsCRP, in patients with CHD or its risk-equivalent diseases receiving intensive atorvastatin therapy [11]. In addition, the level of Lp-PLA2 is significantly reduced with lifestyle improvement (physical activity and dietary moderation) [12] or combined with lipid-lowering therapy [13]. Moreover, the level of Lp-PLA2 is negatively correlated with 6-min walk test (6-MWT) and suitable for assessing exercise tolerance in patients with chronic obstructive pulmonary diseases [14]. There are several ways to develop exercise prescription according to the intensity of exercise, such as heart rate reserve, anaerobic threshold, peak oxygen consumption, target heart rate, and fatigue degree of self-awareness. And 6-MWT, treadmill exercise and bicycle exercise are alternative ways to evaluate exercise intensity. Although treadmill test and bicycle test would be more definite assessment exercise intensity, they need expensive equipments, the higher requirements on operators and pose higher risks to patients. In comparison to the nontrivial costs and logistical challenges of treadmill or bicycle test, a 6-MWT is significantly less expensive and more convenient [15, 16]. Moreover, 6-MWT is a less risky and simpler method pertain to evaluating sub-maximal cardiopulmonary function and maximal oxygen consumption [17, 18].The operating procedure of the 6-MWT only involves simple and cheap equipments, and it’s easy to learn and implement for primary medical institutions. We hypothesized that exercise rehabilitation guided by 6-MWT can effectively improve systemic inflammation in patients with CHD undergoing percutaneous coronary intervention (PCI). In this study, we observed the benefit of 12-week exercise rehabilitation guided by 6-MWT on the Lp-PLA2 level.

## Methods

### Patients and protocols

This was a single-center, prospective, randomized controlled, open clinical trial designed to observe the benefit of exercise rehabilitation guided by 6-MWT on the level of Lp-PLA2 in patients with CHD undergoing PCI. From Dec 2018 to Dec 2020, 100 patients with CHD undergoing PCI were recruited in the fourth ward of the Department of Cardiology, Yuebei People's Hospital Affiliated to Shantou University. Patients were included if they were equal or over 18 years old, with any CHD undergoing PCI, including stable angina and acute coronary syndrome. Patients were excluded if they had respiratory diseases such as chronic obstructive pulmonary disease, obvious abnormal liver and kidney function, New York heart function class III–IV, uncontrolled hypertension or type-2 diabetes mellitus (T2DM), or arrhythmia, unable to cooperate with movement, intolerance for aerobic exercise training etc. This study complied with the principles of the Declaration of Helsinki and was approved by the Ethics Committee of Yuebei People's Hospital Affiliated to Shantou University. All patients had signed the informed consent form. All patients were given optimized drug therapy [19]. The patients were divided into 2 groups using random number table method of simple randomization allocation: Group A, 50 cases, routine health education was given, and the exercise mode and intensity were decided by the patients themselves; and Group B, 50 cases, routine health education and their exercise prescription was guided by 6-MWT. All patients were followed up for 12 weeks.

### Data collection

The baseline clinical data were collected, including gender, age, hypertension, type-2 diabetes mellitus (T2DM), body mass index (BMI, BMI = weight/height (m)^2), etc. Fasting blood was drawed from patients’ elbow vein by a heparin lithium anticoagulant tube (5 ml) and an ethylenediamine tetra acetic acid anticoagulant tube (4 ml), and centrifuged 3000 r/min for 10 min (centrifugal radius 10 cm) at room temperature. Blood lipids, included total cholesterol (TC), three acyl glycerin (TG), and apolipoprotein A (ApoA), apolipoprotein B (ApoB), lipoprotein (a) (Lp (a)), low density lipoprotein cholesterol (LDL—C), high density lipoprotein cholesterol (HDL—C), were tested by full-automatic biochemical analysis instrument (Toshiba TBA2000, Japan). LP-PLA2 was detected by immune enhancement turbidimetry according to the Kit instructions supplied by Nanjing Norman Biological Technology Co., Ltd, China. Collecting fasting blood and tests above were repeated at 12-week follow-up.

### Statistical analysis

Data were expressed as the means ± standard deviation for continuous variables with normal distribution or a median (quartile) for continuous variables with the non-normal distribution and as a frequency for categorical variables. The normal distribution variables were analyzed by two independent sample t test. The non-normal distribution variables were analyzed by Mann–Whitney U test. The categorical variables and count variables were compared between the groups using the χ2 test. The linear regression analysis was applied to examine the effect of the observed indicators on the level of Lp-PLA2 after 12 weeks. The indicators of P ≤ 0.1 were gone into multiple factors regression analysis, and the variables of P ≤ 0.05 in multiple factors regression analysis were included in the equation. For all analyses, a P-value < 0.05 was considered statistically significant. All statistical analyses were performed with the use of SPSS software, version 22.0 (IBM Corp, Chicago, IL, USA).

## Results

**Fig. 1:**
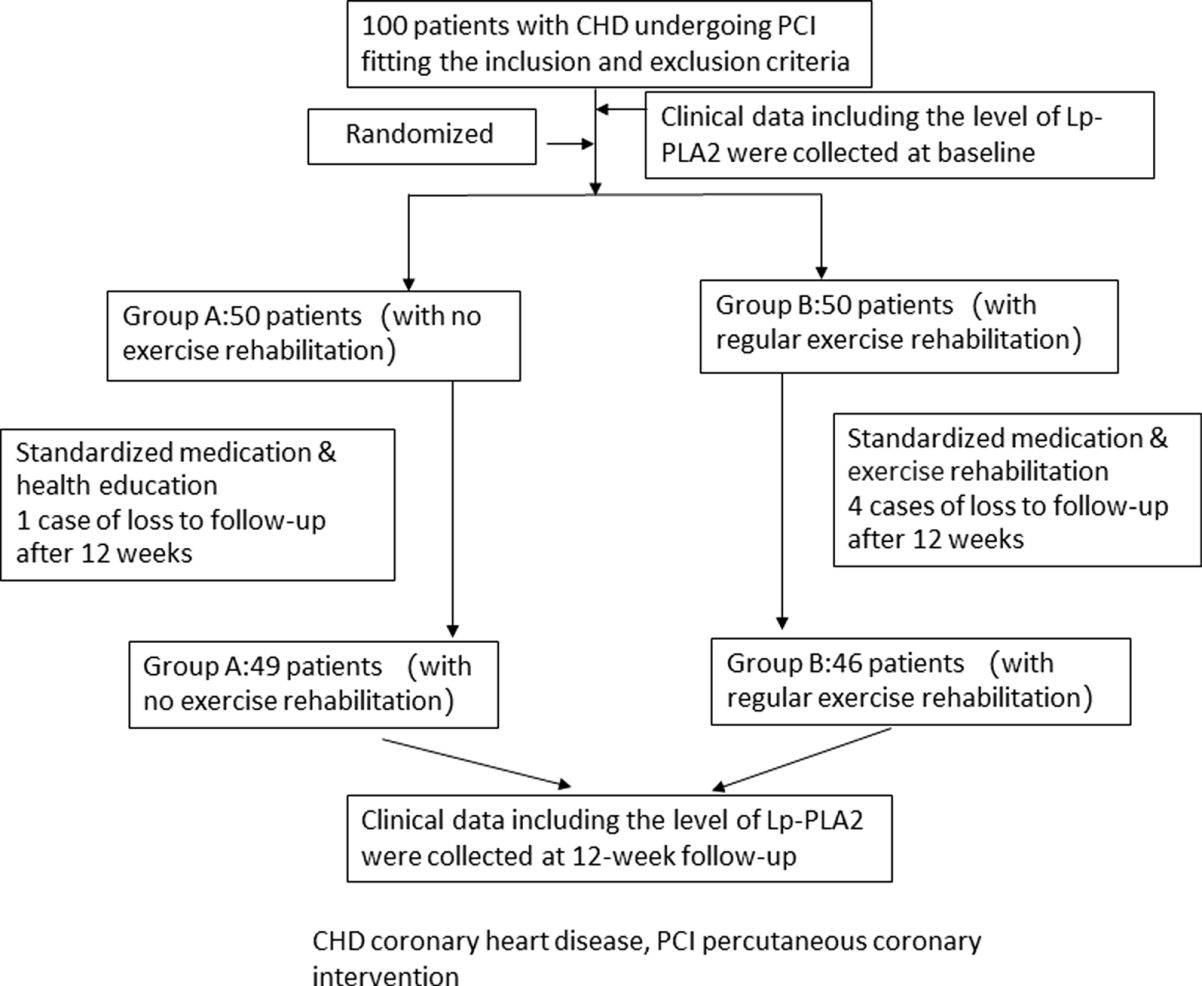
Flowchart of the study

After 12 weeks, there was one case loss to follow-up in Group A and four cases loss to follow-up in Group B, namely, 49 people in group A and 46 people in group B were included in the final analysis (see Fig. 1).

### The comparison of general data between the groups

No statistically significant differences of percentages of gender, hypertension and T2DM between the groups (P > 0.05), however, statistically significant differences of the age were found (t = − 2.552, P = 0.012) (see Table 1).

**Table 1:** Comparison of general information in two groups $\overline{x}$ ± s, N (%)

Group	Group A	Group B	t/χ^2^	P
N	49	46		
Age (years)	59.98 ± 7.69	64.11 ± 8.08^*^	− 2.552	0.012
Male (%)	37 (75.5%)	34 (73.9%)	0.032	0.858
Hypertention (%)	28 (57.1%)	26 (56.5)	3.901	0.272
T2DM (%)	11 (22.4%)	6 (13.0%)	1.429	0.232

T2DM type-2 diabetes mellitus. Compared between Group A and Group B

*P < 0.05

### The comparison of BMI, blood lipids and Lp-PLA2 between the groups

**Fig. 2:**
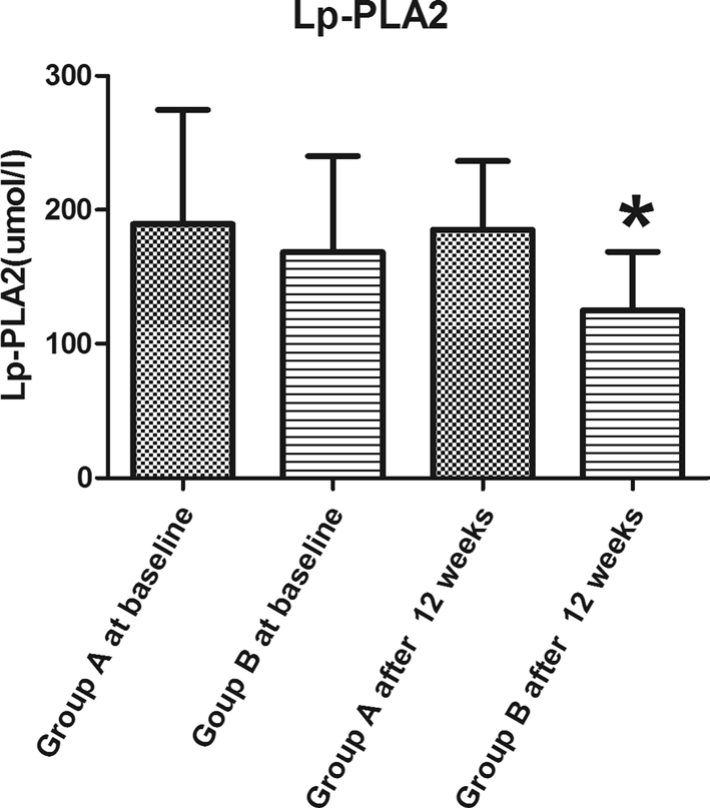
Comparison of Lp-PLA2 between Group A and Group B. Lp-PLA2 = Lipoprotein-associated Phospholipase A2. Compared between group A and group B, ^*^P < 0.05 after 12 weeks

No statistically significant differences of TC, TG, ApoA, ApoB, Lp (a), HDL-C, LDL-C were found between the groups at baseline and at 12 weeks later (P > 0.05, see Table 2). There were no statistically significant differences in the level of Lp-PLA2 between the groups at baseline (P > 0.05, see Table 2). The level of Lp-PLA2 was decreased in two groups after 12 weeks, moreover, the decline of the Lp-PLA2 level in Group B was significantly lower than that in Group A (t = 2.875, P = 0.005, see Table 2 and Fig. 2).

**Table 2:** Comparison of general information between Group A and Group B $\overline{x}$ ± s, M (Q)

Variable		Group A	Group B	t (U) value	P value
N		49	46		
BMI (kg/m^2^)	At baseline	24.04 ± 3.12	23.66 ± 3.76	0.538	0.592
	After 12 weeks	24.23 ± 3.01	23.76 ± 3.70	0.671	0.504
TC (mmol/l)	At baseline	4.52 ± 1.28	4.40 ± 1.02	0.483	0.630
	After 12 weeks	3.80 ± 0.89	3.85 ± 0.84	− 0.299	0.765
TG (mmol/l)	At baseline	1.37 ± 0.70	1.29 ± 0.54	0.598	0.551
	After 12 weeks	1.58 ± 1.03	1.30 ± 0.71	1.508	0.135
Lp (a) (mmol/l)^a^	At baseline	16.90 (36.40)	13.40 (51.56)	976.000	0.780
	After 12 weeks	15.50 (21.90)	13.94 (32.05)	1057	0.854
ApoA (g/l)	At baseline	1.31 ± 0.33	1.35 ± 0.26	− 0.711	0.479
	After 12 weeks	1.37 ± 0.29	1.41 ± 0.28	− 0.758	0.451
ApoB (g/l)	At baseline	0.85 ± 0.23	0.86 ± 0.25	− 0.031	0.976
	After 12 weeks	0.70 ± 0.20	0.70 ± 0.21	− 0.030	0.976
HDL-C (mmol/l)	At baseline	1.13 ± 0.29	1.17 ± 0.23	− 0.672	0.503
	After 12 weeks	1.20 ± 0.29	1.27 ± 0.33	− 1.11	0.270
LDL-C (mmol/l)	At baseline	2.83 ± 1.09	2.75 ± 0.88	0.376	0.708
	After 12 weeks	2.22 ± 0.69	2.08 ± 0.64	1.063	0.291
LP-PLA2 (umol/l)	At baseline	249.58 ± 129.24	221.37 ± 148.8	0.976	0.332
	After 12 weeks	219.13 ± 117.70	155.87 ± 93.80^*^	2.875	0.005

^a^Median and quartile. M (Q) median (quartile), BMI body mass index, TC total cholesterol, TG three acyl glycerin, ApoA apolipoprotein A, ApoB apolipoprotein B, Lp (a) lipoprotein (a), LDL-C low density lipoprotein cholesterol, HDL-C high density lipoprotein cholesterol, LP-PLA2 Lipoprotein-associated Phospholipase A2. Compared between Group A and Group B

*P < 0.05

### The linear regression analysis of the Lp-PLA2 level on general data, BMI, blood lipid and exercise rehabilitation in two groups after 12 weeks

The linear regression results of Lp-PLA2 (as the dependent variable (y)) on general data, BMI, blood lipids and exercise rehabilitation in two groups after 12 weeks were shown in Table 3. The indicators of P ≤ 0.1 were went into multiple factors regression analysis. Finally, exercise rehabilitation (X1), gender (X2), TG (X3), LDL—C (X4) were gone into multiple factors regression analysis. The multiple factors regression analysis of Lp-PLA2 and selected indicators were shown in Table 4. The regression equation was y = 154.327–0.258 X1 + 0.182 X2 + 0.094 + 0.120 X4. That is to say, exercise rehabilitation could reduce the Lp-PLA2 level in CAD patients (β = 46.321, SE = 22.493, β′ = − 0.258, t = − 2.542, P = 0.013).

**Table 3:** The single factor linear regression analysis of the Lp-PLA2 level in group B after 12 weeks

Independent variable	Constant	β	SE	β′	t	P
Exercise rehabilitation	282.383	− 63.258	22.164	− 0.287	− 2.854	0.005
Age (years)	149.008	0.637	1.426	0.047	0.447	0.656
Male (%)	114.712	58.665	25.696	0.233	2.283	0.025
Hypertention (%)	178.358	8.006	9.118	0.092	0.878	0.382
T2DM (%)	190.118	− 8.765	29.899	− 0.031	− 0.293	0.77
BMI (kg/m^2^)	168.392	0.837	3.324	0.026	0.245	0.807
TC (mmol/l)	125.194	16.012	13.559	0.124	1.181	0.241
TG (mmol/l)	152.203	23.698	12.832	0.192	1.847	0.068
Lp (a) (mmol/l)	176.695	0.34	0.345	0.104	0.984	0.328
ApoA (g/l)	237.693	− 36.466	41.367	− 0.093	− 0.882	0.38
ApoB (g/l)	148.409	54.909	57.794	0.1	0.95	0.345
HDL-C (mmol/l)	215.76	− 23.485	38.042	− 0.065	− 0.617	0.539
LDL-C (mmol/l)	115.75	32.983	17.461	0.197	1.889	0.062

Lp-PLA2 Lipoprotein-associated Phospholipase A2, T2DM type-2 diabetes mellitus, BMI body mass index, TC total cholesterol, TG three acyl glycerin, ApoA apolipoprotein A, ApoB apolipoprotein B, Lp (a) lipoprotein (a), LDL-C low density lipoprotein cholesterol, HDL-C high density lipoprotein cholesterol

**Table 4:** The multiple factors regression analysis of the Lp-PLA2 level in group B after 12 weeks

Independent variable	Constant	β	SE	β′	t	P
Exercise rehabilitation	154.327	46.321	22.493	− 0.258	− 2.542	0.013
Male (%)		− 57.186	26.15	0.182	1.771	0.08
TG (mmol/l)		11.61	12.996	0.094	0.893	0.374
LDL-C (mmol/l)		20.093	17.321	0.12	1.16	0.249

Lp-PLA2 Lipoprotein-associated Phospholipase A2, TG three acyl glycerin, LDL-C low density lipoprotein cholesterol

## Discussion

In this study, we demonstrated that the exercise rehabilitation for 12 weeks guided by 6-MWT could further reduce the Lp-PLA2 level in patients with CHD undergoing PCI, which could suggest the exercise rehabilitation for 12 weeks guided by 6-MWT effectively reduce the level of systemic inflammation [11], and thus contribute to the secondary prevention of CHD [8–10].

Cardiac rehabilitation (CR) is a comprehensive intervention measure in chronic stage of patients with cardiovascular disease, which includes drug treatment, exercise rehabilitation, nutrition prescription, psychological prescription and sleep management, no smoking and limited alcohol, and exercise rehabilitation is its core [20]. The exercise prescription of appropriate intensity is the basis of exercise rehabilitation. If the exercise intensity is low, it will be ineffective. However, if exercise intensity is too high, on the one hand, the patient can't insist, on the other hand, patients may be posed higher risks of myocardial ischemia, arrhythmia, heart failure, etc. So we should assess the patients' exercise ability and make appropriate exercise prescription to guide patients' exercise rehabilitation.

Previous studies have showed that the 6-MWT is a simple and valid test for assessing cardiopulmonary fitness [21], is helpful to evaluate overall cardiopulmonary function in elderly patients with CHD [22], and can predict maximal oxygen consumption [18, 23–25]. The 6-MWT could be used in exercise intensity prescription for patients after coronary artery bypass graft surgery, and did not induce hyperlactatemia [26]. At the same time, we can implement 6-WMT only with blood pressure meter and finger-clip oximeter for monitoring the blood oxygen. Therefore, 6-WMT is expected to become a primary medical method for making aerobic exercise prescription in primary medical institutions, which can improve the compliance of home-based exercise rehabilitation. Aerobic training can not only improve the arterial stiffness in older adults [27], older hypertensive patients [28], CHD patients [29], but also reduce the inflammation in patients with CHD [4, 5], while Lp-PLA2 can mediate inflammation [6] which lead to unstable plaque or acute thrombosis.

In order to observe the benefit on Lp-PLA2 of exercise rehabilitation guided by 6-MWT in patients with CHD undergoing PCI, the patients were randomly divided into the exercise rehabilitation group and no-exercise rehabilitation group. No statistically significant differences were found between the groups in gender, hypertension, T2DM, and BMI, blood lipids, and Lp-PLA2 at baseline. The level of Lp-PLA2 decreased at 12-week follow-up, moreover, the decline of the Lp-PLA2 level in exercise rehabilitation group was significant than that in no-exercise rehabilitation group (t = 2.875, P = 0.005). Statistically significant differences of the age were found (t = − 2.552, P = 0.012). Pearson correlation analysis was made to eliminate the effect of the age on the level of Lp-PLA2. Results suggested the differences of the age did not affect the level of Lp-PLA2 (r = 0.035, P = 0.820). In conclusion, this study suggested that the exercise rehabilitation of 12 weeks guided by 6-MWT could further reduce the level of Lp-PLA2 in patients with CHD undergoing PCI, and it was an independent affecting factor of the Lp-PLA2 level. Combining with previous studies and the results of this study, we hypothesized that exercise rehabilitation of 12 weeks guided by 6-MWT could improve the prognosis through reducing the Lp-PLA2 level which could decrease the inflammation in patients with undergoing PCI.

There were some limitations in this study. First, this was a single-center study with relatively small sample size. There might be selection bias even if we enrolled consecutive patients, and there were age differences between groups. However, no significance correlation has been indicated between the age and the level of Lp-PLA2. Second, all patients after PCI were given continuous dual antiplatelet therapy (DAPT) including aspirin and P2Y12 inhibitor, however, the optimal duration of dual antiplatelet therapy (DAPT) after PCI remained uncertain because of the balance between ischemic risk and bleeding risk [30]. Valgimigli et al. found that P2Y12 inhibitor monotherapy was associated with a similar risk of death, myocardial infarction, or stroke, with evidence that this association may be modified by gender, and a lower bleeding risk compared with DAPT [31]. Compared with DAPT, whether P2Y12 inhibitor monotherapy leads to variable Lp-PLA2 needs to be further studied. Third, the patients in the study suffered from different diseases such as stable angina, unstable angina, non-ST-elevation myocardial infarction and ST-elevation myocardial infarction, which may exhibit different profiles of Lp-PLA2. This suggested that there was a need to enlarge the sample size and perform subgroup analysis in order to determine whether exercise rehabilitation guided by 6-min walk test played different roles in the changes of Lp-PLA2 of different types of CHD patients. Finally, we didn’t investigate the impact of the decreased level of Lp-PLA2 on patients’ clinical events, such as angina pectoris, heart failure and arrhythmia, etc. These will be answered by our further outcome research.

## Conclusions

In summary, our study has demonstrated that the exercise rehabilitation for 12 weeks guided by 6-MWT can further reduce the LP-PLA2 level in patients with CHD undergoing PCI, and this scheme of exercise rehabilitation is worth popularizing in primary medical institutions.

## Data Availability

The datasets used in this study are available from the corresponding author on reasonable request.

## Abbreviations

Lp-PLA2: Lipoprotein-associated phospholipase A2
6-MWT: 6-min walk test
CHD: Coronary heart disease
PCI: Percutaneous coronary intervention
T2DM: Type-2 diabetes mellitus
BMI: Body mass index
TC: Total cholesterol
TG: Three acyl glycerin
ApoA: Apolipoprotein A
ApoB: Apolipoprotein B
Lp (a): Lipoprotein (a)
LDL-C: Low density lipoprotein cholesterol
HDL-C: High density lipoprotein cholesterol
M (Q): Median (quartile)
